# Differences in the population structure of *Neisseria meningitidis* in two Australian states: Victoria and Western Australia

**DOI:** 10.1371/journal.pone.0186839

**Published:** 2017-10-24

**Authors:** Shakeel Mowlaboccus, Christopher A. Mullally, Peter C. Richmond, Benjamin P. Howden, Kerrie Stevens, David J. Speers, Anthony D. Keil, Ottar N. Bjørnstad, Timothy T. Perkins, Charlene M. Kahler

**Affiliations:** 1 Marshall Center for Infectious Disease Research and Training, School of Biomedical Sciences, The University of Western Australia, Perth, Western Australia, Australia; 2 Division of Paediatrics, School of Medicine, The University of Western Australia, Perth, Western Australia, Australia; 3 Telethon Kids Institute, Perth, Western Australia, Australia; 4 Microbiological Diagnostic Unit Public Health Laboratory, Department of Microbiology and Immunology, University of Melbourne at The Doherty Institute for Infection and Immunity, Melbourne, Victoria, Australia; 5 Department of Infectious Diseases, Austin Health, Heidelberg, Victoria, Australia; 6 Department of Microbiology, QEII Medical Centre, PathWest Laboratory Medicine WA, Nedlands, Western Australia, Australia; 7 School of Medicine and Pharmacology, The University of Western Australia, Perth, Western Australia, Australia; 8 Department of Microbiology, Princess Margaret Hospital for Children, PathWest Laboratory Medicine WA, Perth, Australia; 9 Center for Infectious Disease Dynamics, The Pennsylvania State University, University Park, Pennsylvania, United States of America; RIVM, NETHERLANDS

## Abstract

*Neisseria meningitidis* is the causative agent of invasive meningococcal disease (IMD). A recombinant vaccine called Bexsero^®^ incorporates four subcapsular antigens (fHbp, NHBA, NadA and PorA) which are used to assign a Bexsero^®^ antigen sequence type (BAST) to each meningococcal strain. The vaccine elicits an immune response against combinations of variants of these antigens which have been grouped into specific BAST profiles that have been shown to have different distributions within geographical locations thus potentially affecting the efficacy of the vaccine. In this study, invasive meningococcal disease isolates from the western seaboard of Australia (Western Australia; WA) were compared to those from the south-eastern seaboard (Victoria; VIC) from 2008 to 2012. Whole-genome sequencing (WGS) of 131 meningococci from VIC and 70 meningococci from WA were analysed for MLST, FetA and BAST profiling. Serogroup B predominated in both jurisdictions and a total of 10 MLST clonal complexes (cc) were shared by both states. Isolates belonging to cc22, cc103 and cc1157 were unique to VIC whilst isolates from cc60 and cc212 were unique to WA. Clonal complex 41/44 represented one-third of the meningococcal population in each state but the predominant ST was locally different: ST-6058 in VIC and ST-146 in WA. Of the 108 BAST profiles identified in this collection, only 9 BASTs were simultaneously observed in both states. A significantly larger proportion of isolates in VIC harboured alleles for the NHBA-2 peptide and fHbp-1, antigenic variants predicted to be covered by the Bexsero^®^ vaccine. The estimate for vaccine coverage in WA (47.1% [95% CI: 41.1–53.1%]) was significantly lower than that in VIC (66.4% [95% CI: 62.3–70.5%]). In conclusion, the antigenic structure of meningococci causing invasive disease in two geographically distinct states of Australia differed significantly during the study period which may affect vaccine effectiveness and highlights the need for representative surveillance when predicting potential impact of meningococcal B vaccines.

## Introduction

Invasive meningococcal disease (IMD) is caused by the Gram-negative diplococcus *Neisseria meningitidis* (the meningococcus), an obligate human bacterium which cause both epidemic and endemic disease around the world [1]. The disease manifests rapidly as septicaemia and/or meningitis and may lead to death within hours [2]. Although IMD is rare, it remains a significant public health concern due to the high mortality and morbidity rate associated with the disease globally [3]. In Australia, the notification rate of IMD is currently 1.1 per 100,000 population although this ratio differs for the eight Australian states ranging from 0.5 to 1.3 per 100,000 population.

**Fig 1:**
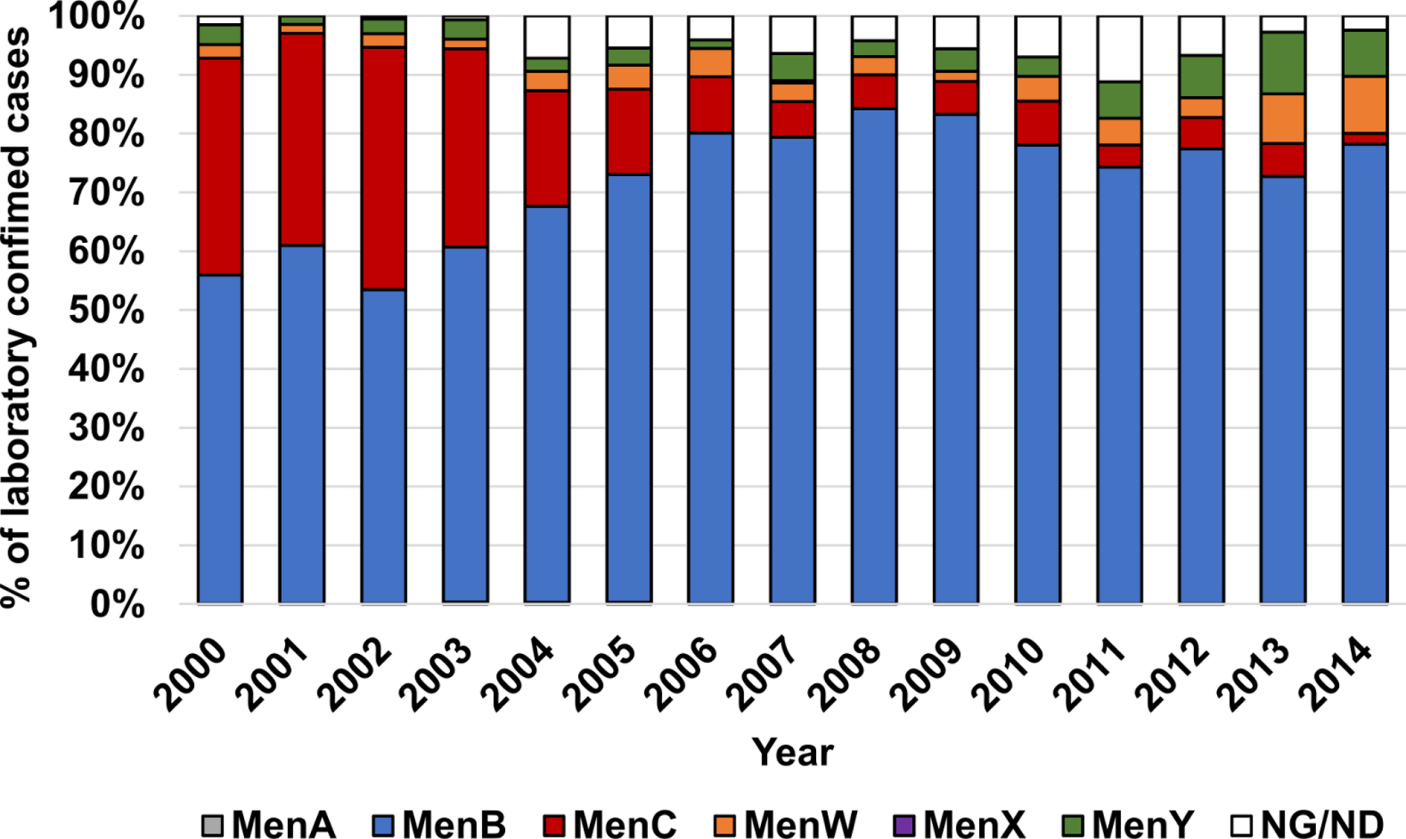
Annual distribution of capsular serogroups expressed by meningococci isolated from laboratory-confirmed IMD cases in Australia. The data for this figure were obtained from the annual reports of the Australian meningococcal surveillance programme (2000–2014). The ‘NG/ND’ category includes meningococci that were not viable for serogrouping or not serogroupable. Of the 4144 meningococcal strains isolated over the 15-year period shown, only 3 MenA (one each in 2003, 2004 and 2005) and 1 MenX isolates (in 2007) were identified. MenB was responsible for more than half of the cases in any year and a decrease in MenC prevalence was observed post-2003.

Meningococci expressing capsule serogroups A, B, C, W, X or Y are more frequently associated with invasive disease than acapsulate strains [4–6] and the distribution of serogroups fluctuates geographically [7]. Conjugate and polysaccharide vaccines providing protection against meningococcal serogroups A, C, W and Y are available. In Australia, since the implementation of the Meningococcal C (MenC) conjugate vaccine on the National Immunisation Programme in 2003, there has been a significant decline in the proportion of IMD caused by serogroup C nationally. Serogroup B has been responsible for ~80% of the annual cases from 2004 to 2014 (Fig 1). Because serogroup B polysaccharide mimics human antigens resulting in poor immunological responses, subcapsular protein-based vaccines against this serogroup have now been developed. The recombinant vaccines being used in the United States and the United Kingdom are Trumenba^®^ [8] and Bexsero^®^ [9], respectively.

The Bexsero^®^ vaccine was made available on the Australian private market in 2013, registered for use in individuals aged 2 months or older, but is not currently part of the national immunisation schedule. The Bexsero^®^ vaccine incorporates one variant each of four sub-capsular antigens: factor H binding protein (fHbp), *Neisseria* adhesin A (NadA), Neisserial Heparin-Binding Antigen (NHBA) and Porin antigen A (PorA) [10]. Variation in the structure and expression of these sub-capsular antigens amongst strains means the vaccine will not elicit immunity against all meningococcal strains. Vaccine coverage of Bexsero^®^, estimated by the monoclonal antibody based meningococcal antigen typing system (MATS) assay, has been shown to vary widely (66–91%) from country to country [11]. In the MATS assay, expression of the fHbp, NadA and NHBA antigens is compared to a minimum threshold of reactivity whilst the PorA antigen is genotyped by PCR and assumed to be covered if the variant belongs to the P1.4 subfamily. The Bexsero^®^ antigen sequence type (BAST) scheme has been developed to reliably monitor the prevalence of vaccine antigens in circulating meningococcal strains [12]. Mowlaboccus et al. [13] have recently reported temporal changes in the BAST profiles of meningococci circulating in WA during the 2000–2014 time period. Despite no vaccine selection pressure, these changes were driven by variations in and within the circulating genetic lineages which resulted in variable (44–91%) predicted annual vaccine coverage over this time period.

**Fig 2:**
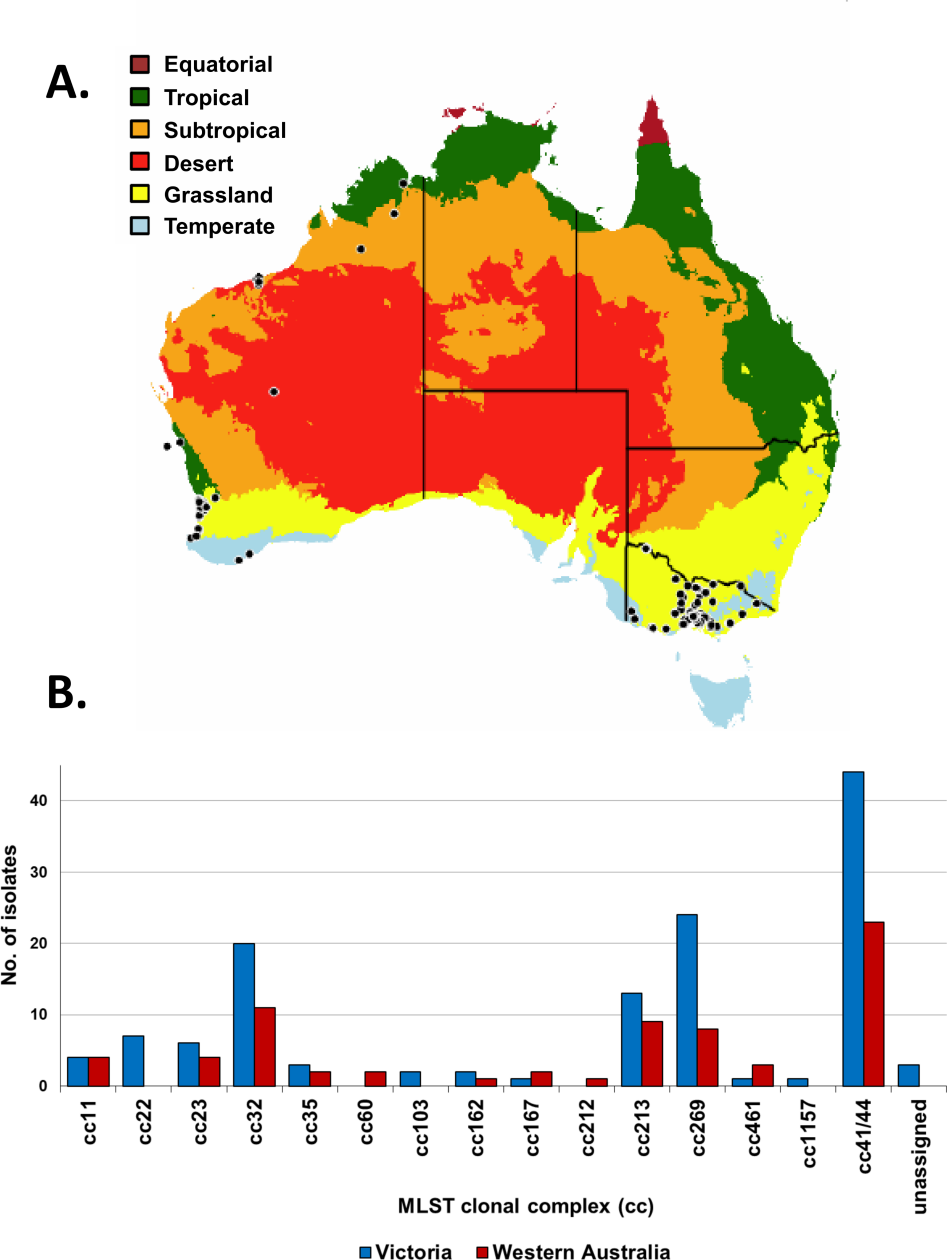
Distribution of MLST clonal complexes in VIC and in WA (2008–2012). (A) WA is located on the western seaboard of Australia whilst VIC is located on the south-eastern seaboard of the continent. Each black dot corresponds to one IMD case and the location is shown based on the postcode provided by the patient. The map is colour-coded based on climatic zones. (B) Analysis of the raw reads from 131 isolates from VIC and 70 isolates from WA identified 79 STs belonging to 15 different clonal complexes (cc) and 3 STs which were not assigned to any known clonal complex.

To determine whether the observations made by Mowlaboccus et al. [13] also apply nationally, the current study compares the population and antigenic structures of meningococci circulating in WA to those in VIC, over a five-year period (2008–12). During this study period, the notification rate of IMD was on average 0.9 and 1.0 per 100,000 population in VIC and WA, respectively. WA is located on the western seaboard of Australia with a land area of 2.6 million square kilometres and a population of ~2 million inhabitants. VIC is located on the south-eastern seaboard of the continent with a land area of 0.24 million square kilometres and a population of ~6 million inhabitants (Fig 2A). Given the restricted number of BAST profiles predicted to be covered by the Bexsero^®^ vaccine, this study is important in evaluating differences, if any, in the meningococcal population of these two Australian states and elucidate whether a vaccine such as Bexsero^®^ should be implemented nation-wide or at the state level.

## Methods

### Bacterial strains and growth conditions

A total of 201 meningococcal strains isolated from disease cases were used in this study– 131 from VIC and 70 from WA (Fig 2). Isolates were passaged fewer than 5 times and were cultured under aerobic conditions with 5% CO_2_ at 37°C on GC agar (Oxoid) supplemented with 0.4% glucose, 0.01% glutamine, 0.2 mg/L of cocarboxylase and 5 mg/L of iron(III) nitrate. Serogroup-specific antisera were used to determine the serogroup of each isolate using slide agglutination.

### Whole-genome sequencing

Genomic DNA extraction was performed using the DNeasy Blood and Tissue Kit (Qiagen, 69506) and stored as per the manufacturer’s instructions for DNA purification from Gram-negative bacteria. Extracted DNA was quantified using Qubit^TM^ fluorimetry and 1.0 ng of DNA was used as input to the Illumina Nextera XT library preparation protocol. The genomic DNA was tagmented, indexed by PCR and purified using AMPure XP beads. The “Library Normalization” step was omitted and the library pooling was performed after quantification of the library size using the LabChip^®^ GXII service provided by the Australian Genome Research Facility. The pooled DNA library which contained genomic DNA (12 pM) from 30 meningococcal strains was loaded onto the Miseq and paired-end 250 bp reads were generated.

### Sequence analysis for MLST and antigen profiling

Sequence analyses were performed according to the analyses used by Mowlaboccus et al. [13]. Briefly, the SRST2 pipeline [14] was used to determine the alleles at the MLST loci (*abcZ*, *adk*, *aroE*, *fumC*, *gdh*, *pdhC* and *pgm*) and the alleles for *fHbp*, *nadA*, *nhba*, *porA* and *fetA*. The nucleotide sequences were then translated and the peptides were assigned integers according to the PubMLST website (https://pubmlst.org/neisseria/)[15]. Phylogenetic trees were constructed using MEGA v7.0 [16]. The BAST profiles were assigned integers according to the classification scheme described by Brehony et al. [12].

### Statistical analysis

Categorical variables were examined by the Chi-squared test. GraphPad Prism 5 (Graph PadSoftware Inc., California) was used to perform the analyses. A 5% level of confidence was used and statistical significance was determined with a p value of < 0.05.

## Results

### Serogroup distribution

Of the 224 notified IMD cases from VIC during 2008–2012, a total of 131 (58%) cases were culture positive and these isolates were sequenced. A total of 70 isolates from Western Australia, which represented 67% of the 104 notified IMD cases during the same time-frame, were also sequenced. Disease pattern with age groups was similar for the two regions during this time period and indicated a period of endemic disease (S1 Fig). No significant difference was observed (*p*>0.05) in serogroup distributions of the sequenced disease-causing isolates from the two states (Table 1). Serogroup B was the most common cause of IMD in both states.

**Table 1:** Serogroup distribution in VIC and WA (2008–2012).

	VIC		WA		
Serogroup	No. of isolates	Proportion	No. of isolates	Proportion	*p* value*
B	111	84.7%	59	84.3%	0.160
C	4	3.1%	5	7.1%	0.115
W	4	3.1%	1	1.4%	0.315
Y	12	9.2%	5	7.1%	0.192
Subtotal	131	100.0%	70	100.0%	

* The *p*-value was calculated using the Fisher’s exact test.

### Distribution of MLST Clonal Complexes

**Fig 3:**
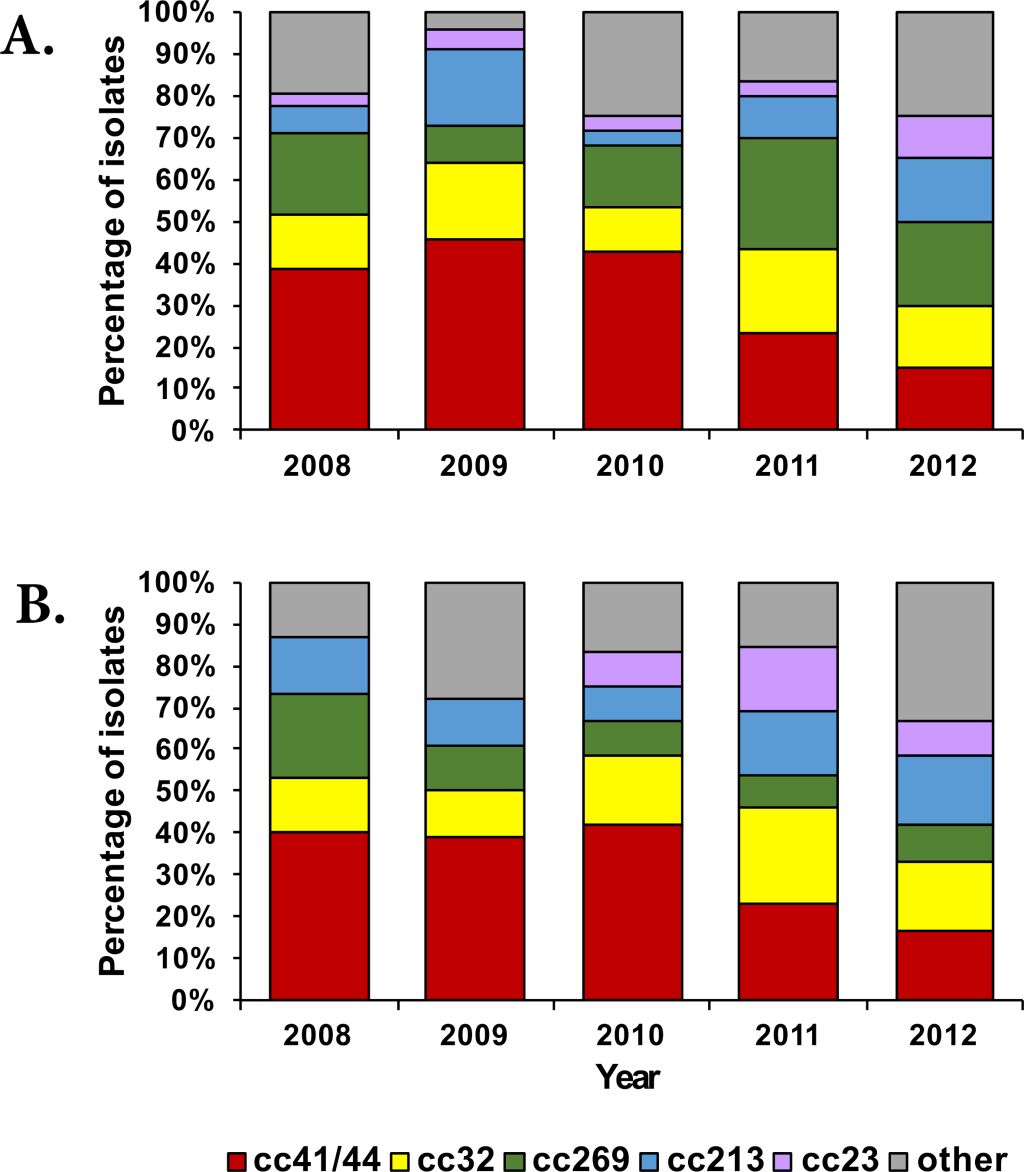
**Clonal complex distribution per annum in VIC (panel A) and WA (panel B) from 2008–2012.** The category “other” indicates clonal complexes with rare frequency (less than 8 isolates) and represents cc11, cc22, cc35, cc60, cc103, cc162, cc167, cc212, cc461, cc1157 and no assigned cc.

A total of 52 STs and 13 clonal complexes (cc) were identified amongst the Victorian isolates. Three of the STs (ST-4267, ST-5118 and ST-novel) were not assigned to any known clonal complex. In the Western Australian collection, a total of 40 STs and 12 clonal complexes were identified. Twelve of the STs identified in this study had not previously been reported globally (11 from VIC and 1 from WA). Isolates belonging to cc22, cc103 and cc1157 were identified in VIC only whilst isolates belonging to cc60 and cc212 were identified in WA only. The two states shared ten clonal complexes (Fig 2B)–cc11, cc23, cc32, cc35, cc162, cc167, cc213, cc269, cc461 and cc41/44. No significant difference was observed in the prevalence of clonal complexes in the two states (S1 Table). The majority of disease in both states was caused by cc41/44 isolates, which represented one-third of the collection in each state (44 isolates in VIC and 23 isolates in WA). However, the predominant ST for this cc was ST-6058 in VIC (n = 11) and ST-146 in WA (n = 7) (S2 Table). A decreasing trend in the annual prevalence of cc41/44 isolates was observed in both states (Fig 3) over the study period. A high proportion of disease in both regions was also caused by isolates belonging to the hypervirulent lineages cc32, cc213 and cc269 with at least 8 isolates identified in each state (S1 Table).

In this study, serogroup C was mainly associated with cc11 and cc212; serogroup W with cc22 and cc167; and serogroup Y with cc22, cc23, cc103 and cc167. The remaining identified lineages were associated with serogroup B. Capsule switching events were observed in cc22 (W→Y) and cc167 (Y→W).

### Distribution of FetA and PorA types

**Fig 4:**
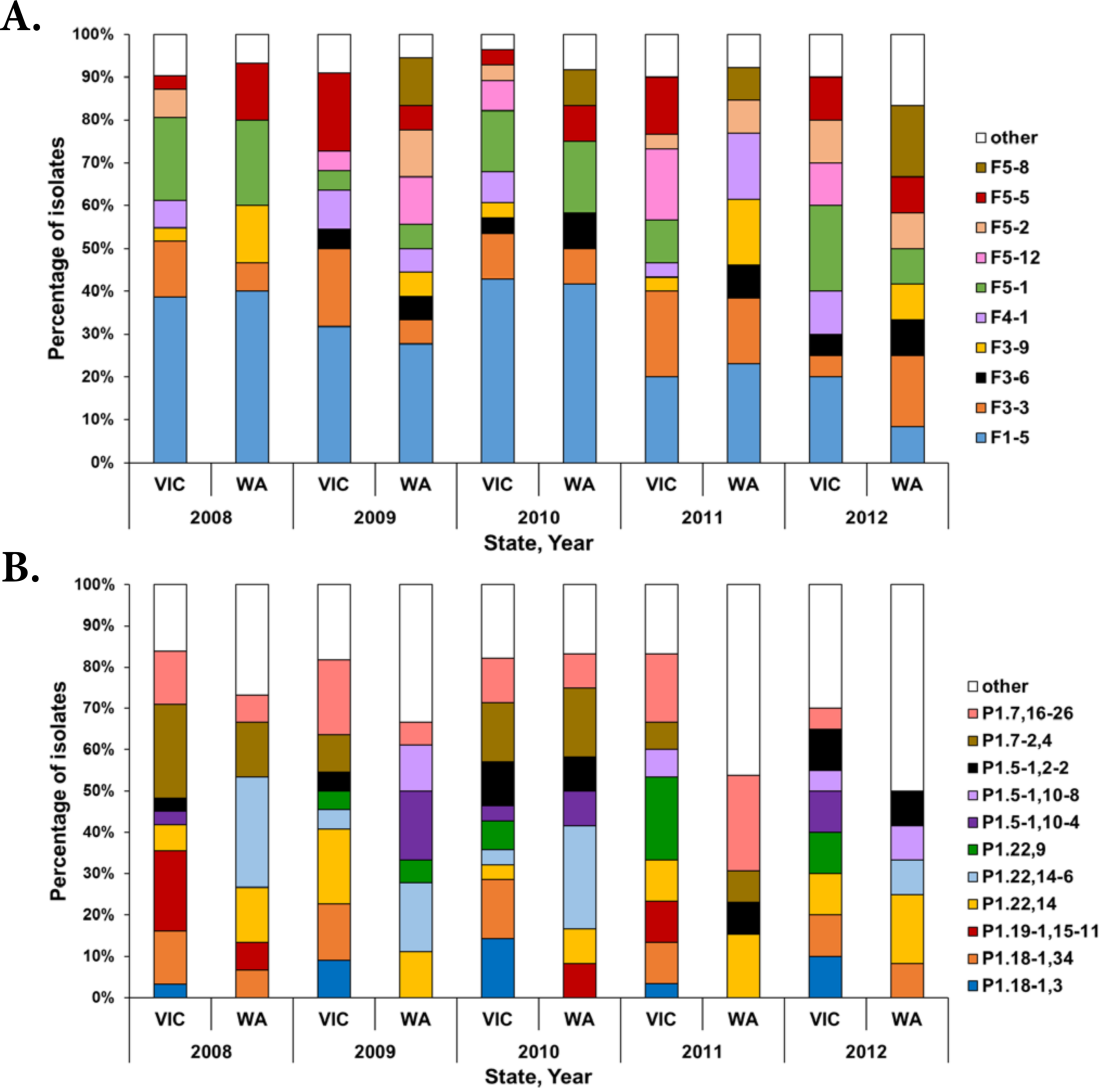
**Distribution of FetA (panel A) and PorA (panel B) types among meningococcal isolates in VIC and WA (2008–2012).** The bars are coloured based on the FetA and PorA types identified in ≥ 5 isolates. The category “other” indicates 12 FetA and 11 PorA types which were observed in < 5 isolates. This analysis includes isolates from 2008, 2009, 2010, 2011 and 2012 from VIC (n = 31, 22, 28, 30 and 20, respectively) and WA (n = 15, 18, 12, 13 and 12, respectively).

A total of 60 *fetA* (NEIS1963) alleles corresponding to 21 FetA types were identified in the entire collection. Eleven FetA types were found in both states. Of the remaining 10, six were unique to VIC and four to WA (Fig 4A). Both states shared the same predominant FetA type, F1-5. In VIC and WA, respectively, 31% (n = 41/131) and 29% (n = 20/70) of isolates possessed alleles for the F1-5 FetA type. Of these 61 isolates, 57 isolates belonged to cc41/44 and the remaining four isolates belonged individually to cc103, cc213, cc269 and to an unassigned cc.

Of the 43 PorA types identified, 15 were found in both states, 15 were identified in VIC only and 13 were identified in WA only (Fig 4B). The most common PorA type in VIC was P1.7,16–26 (Table 2) which was identified in cc32 isolates only. In WA, P1.22,14–6 was the most common PorA type and was identified in cc41/44 isolates only. The P1.7–2,4 PorA type incorporated in the Bexsero^®^ vaccine was identified in 11% (n = 15/131) of VIC isolates and 7% (n = 5/70) of WA isolates. This PorA type was identified in 15 isolates from cc41/44, three isolates from cc11, one isolate from cc32 and one isolate from cc162.

**Table 2:** The predominant antigenic variants of fHbp, NHBA, NadA and PorA identified in VIC and WA (2008–2012).

Australian State	Antigen	No. of unique peptide variants	Most prevalent variant	
			Peptide variant	Number (%)
**VIC** **(n = 131)**	fHbp	30	fHbp-1.4	16 (12)
	NHBA	20	NHBA-2	36 (27)
	NadA	7	no peptide^a^	108 (82)
	PorA	30	P1.7,16–26	17 (13)
**WA** **(n = 70)**	fHbp	22	fHbp-2.19	13 (19)
	NHBA	21	NHBA-3; NHBA-43	11 (16)
	NadA	6	no peptide^a^	58 (83)
	PorA	28	P1.22,14–6	11 (16)

^a^Includes isolates in which the *nadA* gene was either not detected or the gene contained a frame-shift mutation or an insertion element.

A total of 75 PorA:FetA profiles were identified in this collection. The predominant PorA:FetA profile in VIC was P1.7,16–26:F3-3 (n = 17) which was exclusively found in cc32 isolates. The predominant PorA:FetA profile in WA was P1.22,14–6:F1-5 (n = 10) and was exclusively associated with cc41/44 isolates.

### Distribution of the fHbp, NHBA and NadA

Other than the PorA antigen, the Bexsero^®^ vaccine contains 3 additional major surface antigens–fHbp, NHBA and NadA in addition to an outer membrane vesicle component containing many uncharacterised minor antigens.

**Fig 5:**
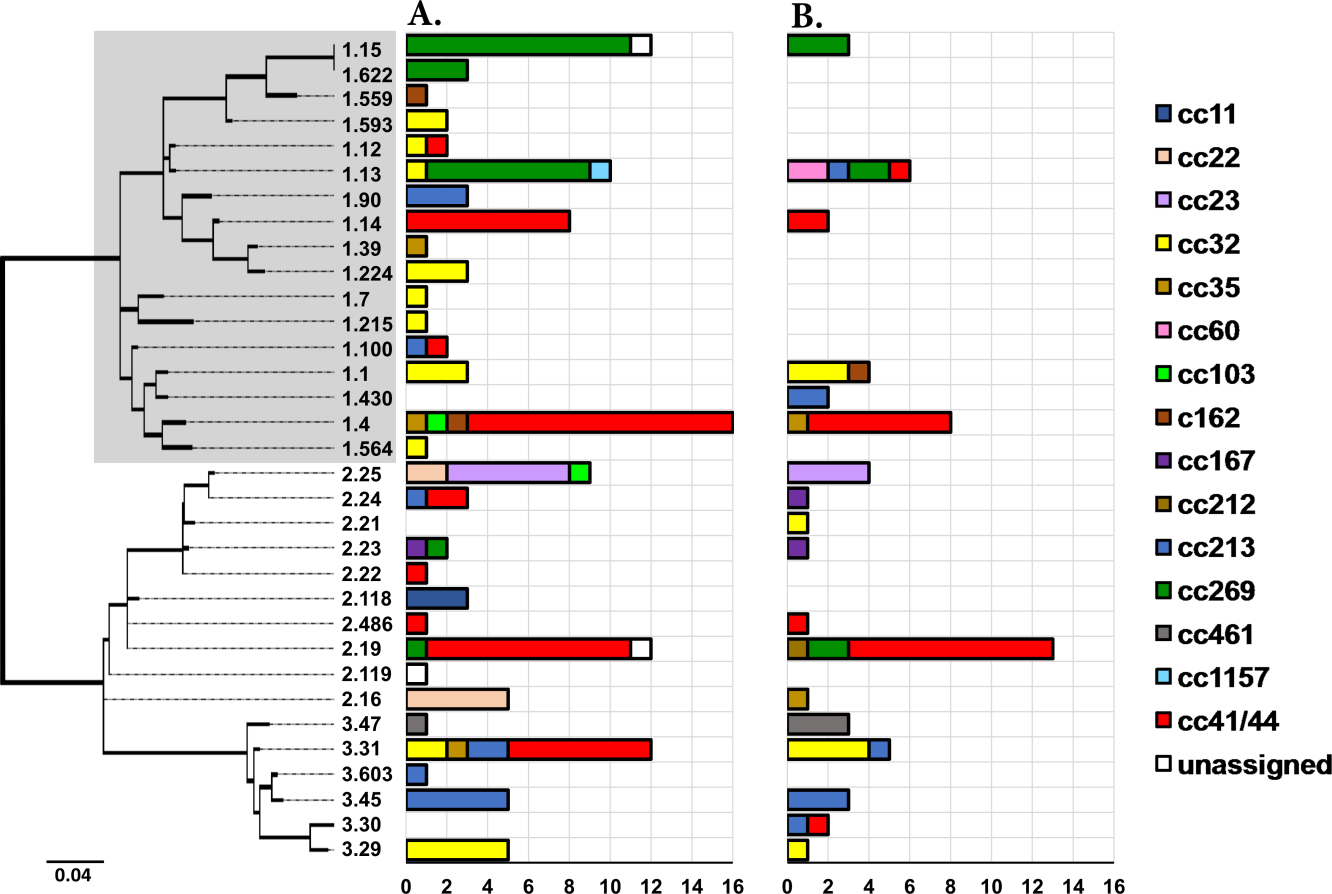
**Distribution of fHbp peptides identified in VIC (panel A) and WA (panel B) during 2008–2012.** The dendrogram represents a maximum likelihood tree (1000 bootstraps) generated using the peptide sequences of 33 fHbp subvariants identified in the collection. Highlighted in grey are the fHbp peptides belonging to the fHbp-1 variant. The distribution of the fHbp peptides is represented in the adjoining bar charts which are coloured by clonal complexes. The fHbp-1.1 peptide is the subvariant incorporated in the Bexsero^®^ vaccine. The dendrogram is drawn to scale, with the sum of the branch lengths between two peptides representing the proportion of amino acid differences between those peptides within the pairwise alignment.

A total of 30 and 18 previously described fHbp peptides were identified in VIC and WA, respectively. Fifteen fHbp peptides were unique to VIC and three fHbp peptides were detected in WA only (Fig 5). The predominant fHbp peptide in VIC was fHbp-1.4 which was identified in 16 isolates–thirteen from cc41/44, one from cc35, one from cc103 and one from cc162. The predominant fHbp peptide in WA was fHbp-2.19 which was identified in 13 isolates–ten from cc41/44, two from cc269 and one from cc212. The fHbp-1.1 peptide incorporated in the Bexsero^®^ vaccine was identified in only seven isolates–three cc32 isolates from each state and one cc162 isolate from WA. Furthermore, the proportion of isolates harbouring alleles for the fHbp-1 variant was significantly lower in WA (*p*<0.05). This difference was particularly noticeable in 2012, when only two isolates from WA compared to eleven isolates from VIC harboured alleles for the fHbp-1 variant (S3 Table).

**Fig 6:**
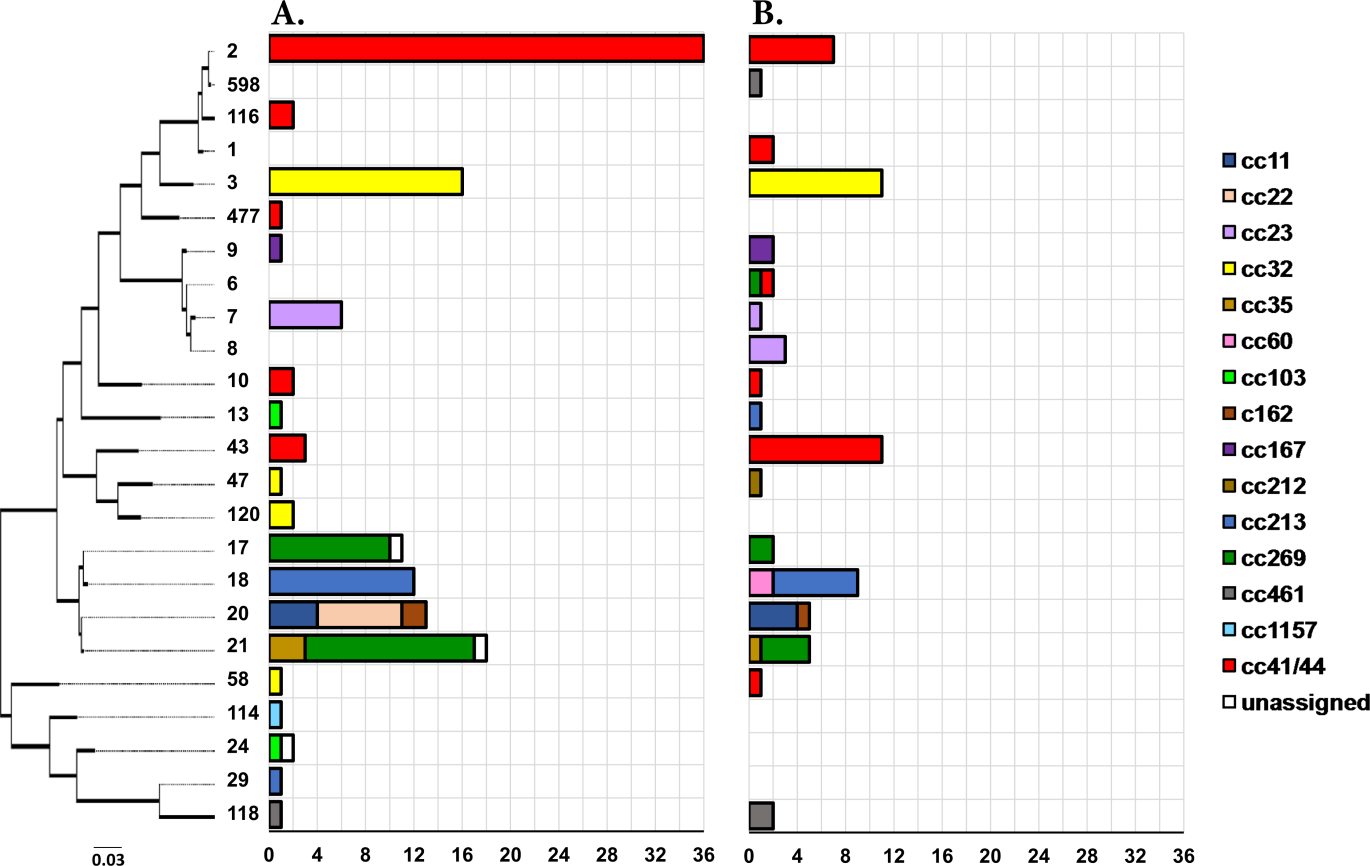
**Distribution of NHBA peptides identified in VIC (panel A) and WA (panel B) during 2008–2012.** The dendrogram represents a maximum likelihood tree (1000 bootstraps) generated using the peptide sequences of the 24 NHBA variants identified in the collection. The distribution of the NHBA peptides is represented in the adjoining bar charts which are coloured by clonal complexes. The NHBA-2 peptide found at the top of the tree is the variant incorporated in the Bexsero^®^ vaccine. The dendrogram is drawn to scale, with the sum of the branch lengths between two peptides representing the proportion of amino acid differences between those peptides within the pairwise alignment.

The *nhba* gene (NEIS2109) was detected in all isolates. A total of 20 and 21 NHBA peptides were identified in VIC and WA, respectively. Fourteen of these peptides were identified in both regions. The NHBA-2 peptide incorporated in the Bexsero^®^ vaccine was the predominant NHBA peptide in VIC and was identified in 27% (n = 36/131) of the Victorian collection. A lower frequency was observed in WA where only 10% (n = 7/70) of isolates harboured genes encoding the NHBA-2 peptide. All isolates possessing alleles for the NHBA-2 peptide were from cc41/44 (Fig 6). NHBA-3 and NHBA-43 were the most common NHBA peptides in WA and were each identified in 11 isolates. All isolates possessing alleles for NHBA-3 and NHBA-43 belonged to cc32 and cc41/44 respectively.

The *nadA* gene (NEIS1969) was absent in all isolates belonging to cc22, cc23, cc35, cc60, cc103, cc162, cc167, cc212, cc269, cc461 and cc41/44. A total of 7 *nadA* alleles were detected in the forty-eight isolates harbouring this gene. These isolates were from cc32 (n = 31), cc11 (n = 8), cc213 (n = 8) and cc1157 (n = 1). The presence or absence of *nadA* correlated with particular lineages with the exception of cc213 where 36% (n = 8/22) harboured this gene whilst the remainder lacked the entire locus. Twenty-seven isolates (26 from cc32 and one from cc11) encoded a *nadA* allele 19, generating a putative atypical protein (NadA-100 peptide) with a longer anchor domain and an altered distal portion [17]. Of these isolates, nineteen were from VIC, including the cc11 isolate, and eight were identified in WA. Four isolates (three from VIC and one from WA), all belonging to cc11:ST-11, possessed a *nadA* allele 29 which contains the insertion sequence IS*1301*, and would consequently be unable to express an intact NadA peptide. Eight isolates (five from VIC and three from WA) harboured *nadA* alleles with internal stop codons and would unlikely be able to express a peptide. Therefore, only nine isolates were considered positive for the NadA antigen. Alleles for the NadA-1.1 peptide were identified in six isolates (two from VIC and four from WA) which were from cc32 (n = 5) and cc213 (n = 1), all expressing a serogroup B capsule. Alleles for the NadA-2/3.2 peptide were identified in the remaining three isolates (two from VIC and one from WA), all of which belonged to cc11 (serogroup C, ST-11).

### Prevalence of the antigenic variants incorporated in the Bexsero^®^ vaccine and BAST distribution

A significantly larger proportion of isolates harbouring alleles encoding the exact antigenic variants for one or more of the four major immunogenic peptides incorporated in the Bexsero^®^ vaccine (fHbp-1.1, NHBA-2, NadA-2/3.8 and P1.7–2,4) was identified in VIC (*p*<0.05) (Table 3). This difference was mainly due to the higher proportion of isolates harbouring alleles for the NHBA-2 peptide observed in VIC compared to WA (*p*<0.01). All isolates harbouring alleles for fHbp-1.1 or NHBA-2 expressed a serogroup B capsule. Of the 20 isolates harbouring alleles for the Bexsero^®^ specific PorA vaccine antigen, seventeen expressed a serogroup B capsule whilst the remaining three isolates expressed a serogroup C capsule. These three isolates, all from VIC, belonged to the ST-11 lineage and were identical at the *fHbp* and *nhba* loci, encoding for fHbp-2.118 and NHBA-20. No isolates harbouring alleles for three or four Bexsero^®^ specific vaccine antigenic variants were observed. One cc32 isolate, from WA, harboured alleles for fHbp-1.1 and P1.7–2,4. Fourteen isolates (11 from VIC and 3 from WA), all belonging to cc41/44, harboured alleles for NHBA-2 and P1.7–2,4. None of the isolates harboured alleles for fHbp-1.1 and NHBA-2 concurrently.

**Table 3:** Prevalence of the exact antigenic variants of fHbp, NHBA, NadA and PorA incorporated in the Bexsero^®^ vaccine.

	All Serogroups			Serogroup B		
Antigen	VIC (n = 131)	WA (n = 70)		VIC (n = 111)	WA (n = 59)	
	Number (%)	Number (%)	*p*-value	Number (%)	Number (%)	*p*-value^b^
**fHbp-1.1**	3 (2)	4 (6)	0.14	3 (3)	4 (7)	0.14
**NHBA-2**	36 (27)	7 (10)	0.002	36 (32)	7 (12)	0.002
**NadA-2/3.8**	0 (0)	0 (0)	1	0 (0)	0 (0)	1
**P1.7–2,4**	15 (11)	5 (7)	0.13	12 (11)	5 (8)	0.19
**Either^a^**	43 (33)	12 (17)	0.008	40 (36)	12 (20)	0.015

^a^Includes isolates possessing alleles for either one or more exact antigenic variants of the vaccine antigens.

^b^The *p*-value was calculated using the Fisher’s exact test.

A total of 66 and 51 BASTs were identified in VIC and WA, respectively. Only nine BASTs were simultaneously observed in the two states. The most common BAST identified in VIC was fHbp-2.19:NHBA-2:NadA-absent:P1.18–1,34 (BAST-315) which was represented by 10 isolates, all of which belonged to cc41/44 (eight ST-6058, one ST-409 and one ST-9991) and expressed a serogroup B capsule. In contrast, the most common BAST identified in WA was fHbp-2.19:NHBA-43:NadA-absent:P1.22,14–6 (BAST-933) which was represented by 8 isolates, all of which belonged to cc41/44 (four ST-146 and two ST-10511) and expressed a serogroup B capsule.

## Discussion

IMD is a notifiable disease in Australia and since 1994, the National Neisseria Network has been supplying information annually on the serogroup, serotype (PorB typing), serosubtype (PorA typing) and antibiotic susceptibility of invasive meningococci isolated from the eight Australian states and territories as part of the Australian Meningococcal Surveillance Programme [18]. Unlike previous epidemiological studies on meningococci circulating around Australia [13, 19–33], this current study is the first to use WGS to compare the MLST and BAST profiles of invasive meningococci from two states found on either side of the Australian continent over the same 5-year period (2008–2012). With the exception of FetA, the typing data used for the WA isolates have previously been published by our group [13].

Serogroup distribution was similar in both states and meningococci expressing serogroup B capsule were predominant in each state. No significant differences were observed for the distribution of clonal complexes between the two states with the exception of cc22 (S1 Table). The seven isolates belonging to cc22 were identified in VIC only and expressed either a serogroup W (n = 4) or a serogroup Y (n = 3) capsule. Furthermore, cc103 and cc1157 were unique to VIC and cc60 and cc212 were unique to WA. However, since less than three isolates belonged to each of these lineages (S1 Table), most likely representing sporadic cases, the exclusiveness of these clonal complexes was not statistically supported (*p*>0.05). Also, the short time-period over which the analysis was conducted was inadequate to corroborate the restriction of a particular clonal complex to one region.

Clonal complex 41/44 was the predominant lineage, representing one-third of the meningococcal population, in each jurisdiction during the analysed time-period but the predominant ST for this lineage was locally different–ST-6058 in VIC and ST-146 in WA. Despite the high prevalence reported in this study, none of these two STs have so far been reported as the causative agent of an IMD outbreak. The PubMLST database contains 9 records of ST-6058 and 11 records of ST-146 associated with invasive disease, most of which were identified in Europe in the 21^st^ century except one ST-146 isolate which caused disease in the Netherlands as early as 1980. Given that ST-146 was already dominant in WA prior to 2008 [13] and since ST-6058 differs from ST-146 at three housekeeping loci (*abcZ* [4 SNPs], *adk* [4 SNPs] and *pdhC* [7 SNPs]), it can be hypothesized that the propagation of these two STs of the same clonal complex occurred in parallel in each region. Longitudinal studies have reported that meningococcal strains of a particular ST can persist in a population for decades [34–36]. However, since the majority of isolates in the PubMLST database have a more recent provenance and this database does not necessarily provide a detailed picture of past events pre-2008 or represent all geographic regions equally well, this remains an untested hypothesis.

In addition to the MLST profiles, the PorA:FetA and BAST profiles identified in this collection were diverse. Only 16% (12/75) of the PorA:FetA and 8% (9/108) of the BAST profiles were shared by the two jurisdictions although no significant difference in clonal complex distribution was observed, suggesting that the antigenic profiles of meningococci circulating in these two states were different during 2008–2012. Differences in the antigenic structure of meningococci may be attributed to differences in the host population. For example, Boan et al. [31] have previously reported an independent association of fHbp-2 with aboriginal cases and hence, the difference in BAST distribution in VIC and WA may be due to the different proportions of aboriginal population inhabiting the two regions (0.9% in VIC vs 3.8% in WA as at June 2011, Australian Bureau of Statistics). In addition to demographic factors, the difference in BAST profiles may also reflect the separate introduction, and successful diversification, of distinct meningococcal clones through international travel in each state.

The dissemination of invasive meningococci within a certain population depends on the survival and effective transmission between human hosts and the ability to become established in the nasopharynx by evading natural immunity. The ability to evade host immunity through ongoing genetic diversification is clearly reflected in the high diversity of BAST profiles detected within a small region [34]. Previous studies, conducted in Europe, have shown that the meningococcal population structure varies geographically and is genetically different even for neighbouring countries [12, 37, 38]. However, this current study shows that, in the case of Australia, no significant difference in the distribution of clonal complexes was observed between VIC and WA despite the relatively larger separation of ~4100 km, when compared to Europe. This may be the result of the shorter time frame of 5 years examined in this study suggesting that a longitudinal study of greater duration is warranted. Furthermore, unlike the study conducted by Abad et al. [37] which showed that variations in predicted strain coverage of Bexsero^®^ in Spain were attributed to the prevalence of clonal complexes, we report that although cc41/44 predominated in both states, the STs and BAST profiles of strains circulating separately in VIC and WA were different. The predominant BAST (BAST-315) in VIC is covered by the NHBA-2 antigen while the predominant BAST (BAST-933) in WA is not covered by any of the four antigens.

Despite considerable diversity in BAST profiles, there is evidence that only one cross-reactive variant from the four vaccine antigens is sufficient to provide immune protection [11]. The presence of at least one of the following antigenic variants proposed by Mowlaboccus et al. [13], derived from the positive results of a MATS ELISA assay, was used to predict the vaccine coverage for the two Australian states: fHbp-1 (except fHbp-1.13), NHBA-1, NHBA-2, NadA-1, NadA-2/3, P1.4 subfamily PorA. Using these parameters, the predicted vaccine coverage in WA (47.1% [95% CI: 41.1–53.1%]) was significantly lower than that in VIC (66.4% [95% CI: 62.3–70.5%]) (*p*<0.05). This difference was mainly due to the higher prevalence of the NHBA-2 variant in meningococcal isolates from VIC (Fig 6) and the smaller proportion of meningococci harbouring the fHbp-1 variant in WA (Fig 5). These estimates of predicted vaccine coverage for these states during the studied period was lower than the national coverage of 76% predicted by the MATS ELISA by Nissen et al. [39] in a study of 373 serogroup B meningococci isolated in Australia during 2007–2011. The difference in predicted coverage is most likely due to the under-representation of Victorian and Western Australian isolates used in the Nissen et al. study [39]. Hence, in the case of Australia, meningococcal STs and BASTs do not necessarily prevail throughout the country over short time frames but may rather be restricted to the western or the eastern seaboard. Future molecular surveillance is required to understand these spatial and temporal changes in meningococcal population diversity across the country over long periods of time to examine whether administering a recombinant vaccine like Bexsero^®^ should be considered at a national level or if this decision should be rather taken at a state level.

Lastly, while outbreaks of serogroup W:cc11 occurred in other parts of the world during the period of this study [40, 41], no such strain was detected until 2013 in either Victoria [42, 43] or Western Australia [13]. Since 2013, there has been an increase in IMD caused by serogroup W all-round the continent which resulted in this serogroup becoming the predominant meningococcal serogroup in Australia in 2016 [44]. Clonal expansion of penicillin-resistant MenW:cc11 isolates harbouring the *penA*_253 allele has recently been reported in WA [45] but this allele was identified in only one isolate (serogroup Y, cc22, VIC) from our collection which was also resistant to penicillin.

Although IMD is rare, disease patterns vary widely not only geographically but also temporally, and therefore ongoing surveillance is of utmost importance to identify the causes of potential outbreaks for rapid intervention. As this study shows that the antigenic structure of *N*. *meningitidis* is different in Victoria and Western Australia, we encourage WGS of meningococci from the other Australian states to better elucidate the molecular epidemiology of strains circulating around Australia before recommending the national implementation of a new meningococcal vaccine.

## Supporting information

S1 Fig Distribution of serogroups among age groups in VIC and WA (2008–12). The bars are coloured based on serogroups.
(TIF)

S1 Table Distribution of clonal complexes in VIC and WA (2008–12). (PDF)

S2 Table Antigenic diversity in meningococcal isolates from VIC and WA (2008–2012). (PDF)

S3 Table Annual prevalence of the fHbp-1 variant in VIC and WA during 2008–2012. (PDF)
