# Supplementary figures and images for: Differences in the population structure of Neisseria meningitidis in two Australian states: Victoria and Western Australia
Source: PLoS One. 2017 Oct 24;12(10):e0186839. doi: 10.1371/journal.pone.0186839 (PMC5655437; doi:10.1371/journal.pone.0186839)

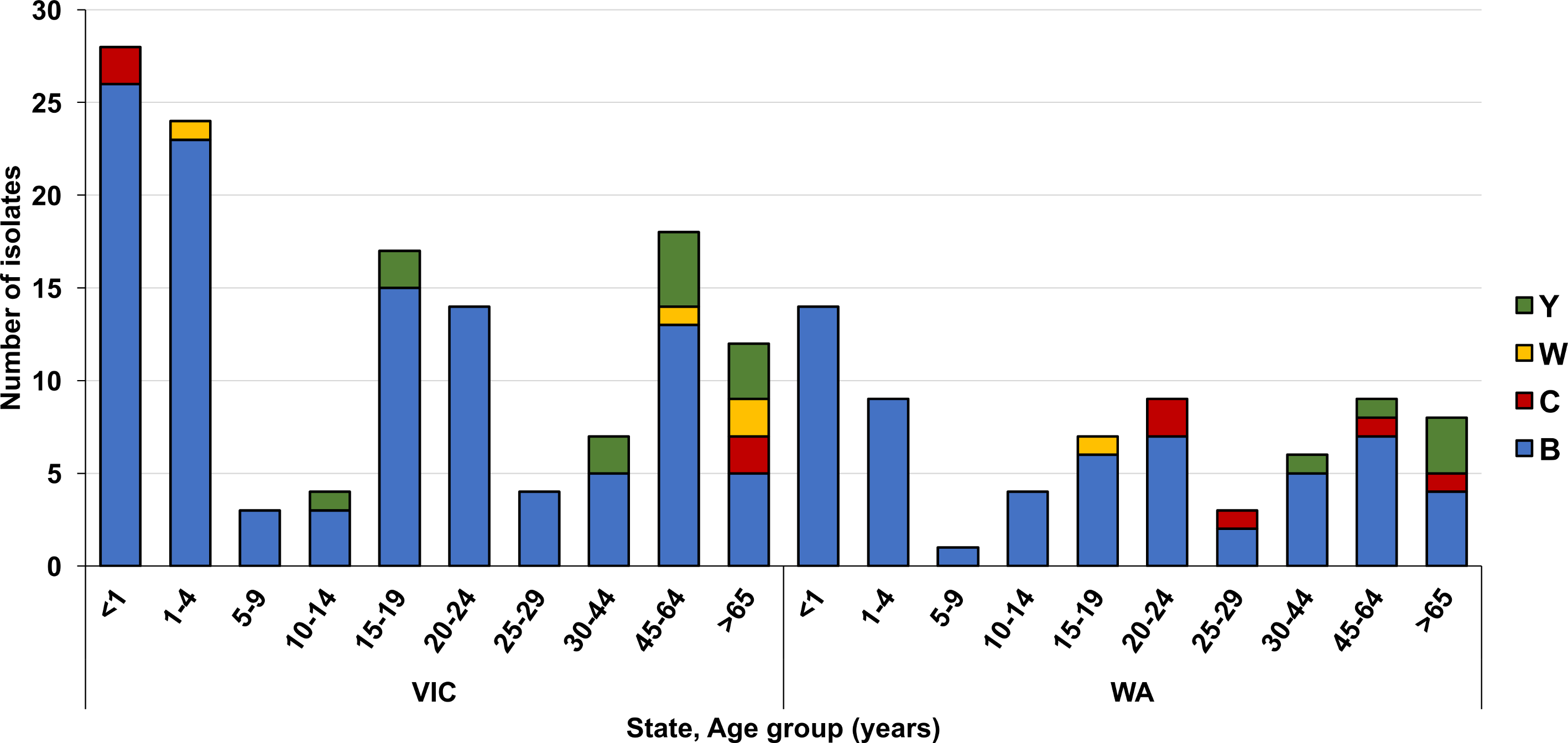

Supplement: S1 Fig — The bars are coloured based on serogroups. (TIF) [file pone.0186839.s001.tif]
