# Supplementary material for: Differences in the population structure of Neisseria meningitidis in two Australian states: Victoria and Western Australia
Source: PLoS One. 2017 Oct 24;12(10):e0186839. doi: 10.1371/journal.pone.0186839 (PMC5655437; doi:10.1371/journal.pone.0186839)
Supplement: S1 Table — (PDF) [file pone.0186839.s002.pdf]

S1 Table. Distribution of clonal complexes in VIC and WA (2008-12)

	VIC		WA		
**Clonal complex**	**No. of isolates**	**Proportion**	**No. of isolates**	**Proportion**	***p* value**[a]
cc11	4	3.1%	4	5.7%	0.1872
cc22	7	5.3%	0	0.0%	0.0472
cc23	6	4.6%	4	5.7%	0.2425
cc32	20	15.3%	11	15.7%	0.1609
cc35	3	2.3%	2	2.9%	0.3400
cc60	0	0.0%	2	2.9%	0.1201
cc103	2	1.5%	0	0.0%	0.4236
cc162	2	1.5%	1	1.4%	0.4470
cc167	1	0.8%	2	2.9%	0.2373
cc212	0	0.0%	1	1.4%	0.3483
cc213	13	9.9%	9	12.9%	0.1495
cc269	24	18.3%	8	11.4%	0.0745
cc461	1	0.8%	3	4.3%	0.1087
cc1157	1	0.8%	0	0.0%	0.6571
cc41/44	44	33.6%	23	32.9%	0.1242
unassigned	3	2.3%	0	0.0%	0.2746
Subtotal	131	100.0%	70	100.0%	

[a]The *p*-value was calculated using the Fisher's exact test.
