# Supplementary material for: Differences in the population structure of Neisseria meningitidis in two Australian states: Victoria and Western Australia
Source: PLoS One. 2017 Oct 24;12(10):e0186839. doi: 10.1371/journal.pone.0186839 (PMC5655437; doi:10.1371/journal.pone.0186839)
Supplement: S2 Table — (PDF) [file pone.0186839.s003.pdf]

S2 Table. Antigenic diversity in meningococcal isolates from VIC and WA (2008-2012)

**Genotype**	**No. of molecular types**			**Most common molecular type**	
	**VIC (n=131)**	**WA (n=70)**	**Entire collection (n=201)**	**VIC**	**WA**
**Clonal complex**	13	12	15	cc41/44	cc41/44
**ST**	52	40	82	ST-6058	ST-146
**FetA:**					
alleles	37	36	60	allele 20	allele 20
peptides	17	15	21	F1-5	F1-5
**PorA:**					
alleles	34	32	49	allele 241	allele 101
peptides	30	28	43	P1.7,16-26	P1.22,14-6
**PorA:FetA**	44	43	75	P1.7,16-26:F3-3	P1.22,14-6:F1-5
**fHbp:**					
alleles	38	24	40	allele 4	allele 19

mutations*	1	2	3	-	-
peptides	30	22	37	fHbp-1.4	fHbp-2.19
**NHBA:**					
alleles	22	24	32	allele 1	allele 5; allele58
peptides	20	21	27	NHBA-2	NHBA-3; NHBA-43
**NadA:**					
alleles	7	6	7	-	-
mutations	4	3	4	-	-
peptides	3	3	3	-	-
**BAST**	66	51	108	fHbp-2.19:NHBA-2:NadA-absent:P1.18-1,34	fHbp-2.19:NHBA-43:NadA-absent:P1.22,14-6

*****deletion or frameshift mutations
