# Supplementary material for: Differences in the population structure of Neisseria meningitidis in two Australian states: Victoria and Western Australia
Source: PLoS One. 2017 Oct 24;12(10):e0186839. doi: 10.1371/journal.pone.0186839 (PMC5655437; doi:10.1371/journal.pone.0186839)
Supplement: S3 Table — (PDF) [file pone.0186839.s004.pdf]

**S3 Table. Annual prevalence of the fHbp-1 variant in VIC and WA during 2008-2012.**

| | **VIC** | | **WA** | | |
|---|---|---|---|---|---|
| **Year** | **No. of isolates** | **No. of isolates possessing alleles for fHbp-1** | **No. of isolates** | **No. of isolates possessing alleles for fHbp-1** | ***p*-value**[a] |
| **2008** | 31 | 19 | 15 | 5 | 0.054 |
| **2009** | 22 | 13 | 18 | 8 | 0.166 |
| **2010** | 28 | 12 | 12 | 5 | 0.272 |
| **2011** | 30 | 14 | 13 | 6 | 0.260 |
| **2012** | 20 | 11 | 12 | 2 | 0.032 |
| **Total** | 131 | 69 | 70 | 26 | 0.013 |

[a]The *p*-value was calculated using the Fisher's exact test.
